# Gut microbiota and undigested food constituents modify toxin composition and suppress the genotoxicity of a naturally occurring mixture of *Alternaria* toxins in vitro

**DOI:** 10.1007/s00204-020-02831-1

**Published:** 2020-07-04

**Authors:** Francesco Crudo, Georg Aichinger, Jovana Mihajlovic, Luca Dellafiora, Elisabeth Varga, Hannes Puntscher, Benedikt Warth, Chiara Dall’Asta, David Berry, Doris Marko

**Affiliations:** 1 Department of Food Chemistry and Toxicology, Faculty of Chemistry, University of Vienna, Währinger Str. 42, 1090 Vienna, Austria; 2 Department of Food and Drug, University of Parma, Area Parco delle Scienze 27/A, 43124 Parma, Italy; 3 Department of Microbiology and Ecosystem Science, Centre for Microbiology and Environmental Systems Science, University of Vienna, Althanstr. 14, 1090 Vienna, Austria; 4 Joint Microbiome Facility of the Medical University of Vienna and the University of Vienna, Vienna, Austria

**Keywords:** Mold, Microbiome, Chemical mixture, Genotoxicity, Food contaminant, Bacteria

## Abstract

Molds of the genus *Alternaria* produce several mycotoxins, some of which may pose a threat for health due to their genotoxicity. Due to the lack of adequate toxicological and occurrence data, they are currently not regulated. Interactions between mycotoxins, gut microbiota and food constituents might occur after food ingestion, modifying the bioavailability and, therefore, overall toxicity of mycotoxins. The present work aimed to investigate the impact of in vitro short-term fecal incubation on the in vitro DNA-damaging effects exerted by 5 µg/mL of an *Alternaria alternata* extract, containing, among others, 15 nM alternariol, 12 nM alternariol monomethyl ether, 241 nM altertoxin II and 301 nM stemphyltoxin III, all of which are known as genotoxic. The involvement of microorganisms, undigested food constituents and soluble substances of human fecal samples in modifying the composition and the genotoxicity of the extract was investigated through the application of LC–MS/MS analysis and comet assays in HT-29 cells. Results showed that the potential of the mycotoxins to induce DNA strand breaks was almost completely quenched, even before anaerobic incubation, by contact with the different fractions of the fecal samples, while the potency to induce formamidopyrimidine DNA glycosylase (FPG)-sensitive sites was only slightly reduced. These effects were in line with a reduction of mycotoxin concentrations found in samples analyzed by LC–MS/MS. Although a direct correlation between the metabolic activity of the gut microbiota and modifications in mycotoxin contents was not clearly observed, adsorptive phenomena to bacterial cells and to undigested food constituents might explain the observed modifications.

**Electronic supplementary material:**

The online version of this article (10.1007/s00204-020-02831-1) contains supplementary material, which is available to authorized users.

## Introduction

*Alternaria* mycotoxins are low-molecular-weight compounds produced by different *Alternaria* species, among which *Alternaria alternata* is the most important mycotoxin-producing species of this genus of molds (EFSA 2011).

*Alternaria* species can infect various fruits and vegetables and can produce, under favorable conditions of temperature and humidity, more than 70 structurally characterized toxins (Ostry 2008; EFSA 2011). Some *Alternaria* mycotoxins are thought to represent a threat for human and animal health since they have shown a wide number of adverse effects in vivo (teratogenic and fetotoxic effects) and in vitro (genotoxic, clastogenic, mutagenic, estrogenic and androgenic effects) (EFSA et al. 2016). Regarding toxicity, alternariol (AOH), alternariol monomethyl ether (AME), altenuene (ALT), tenuazonic acid (TeA) and tentoxin (TEN) are the most studied of these compounds. However, few toxicological and occurrence data are available for other *Alternaria* mycotoxins of potential high relevance for food safety, such as alterperylenol (ALP), altersetin (AST), altertoxins (ATXs) I, II, and III, stemphyltoxin III (STTX-III), altenuisol, altenuic acid and the *A. alternata* f. sp. *lycopersici* toxins (AAL-TA and AAL-TB).

Among the substances which were most frequently reported in food and feed, both AOH and AME were found to act as topoisomerase poisons, leading to single- and double-DNA strand breaks, while ALT and iso-ALT did not show any genotoxic properties (Fehr et al. 2009). The ability of AOH and AME to poison topoisomerases is thought to be a consequence of their planar structure, which would favor DNA pair-base intercalation (Dellafiora et al. 2015). Interestingly, the perylene quinones ATX-II and STTX-III have been described to be even more genotoxic than AOH, acting probably through the formation of mycotoxin-DNA adducts as the main mechanism (Schwarz et al. 2012b; Fleck et al. 2016). Besides the genotoxic effects, AOH and AME have also been reported to act as possible endocrine disruptors (Frizzell et al. 2013). As a matter of fact, both mycotoxins have been found to activate estrogen receptors (Lehmann et al. 2006; Dellafiora et al. 2018), and AOH can act as a full androgen agonist (Stypuła-Trębas et al. 2017).

Despite the potential threat to human and animal health posed by some *Alternaria* mycotoxins, no specific regulations are currently in place. In this context, the risk assessment on chemical contaminants is currently based on the evaluation of the effects of the single substances, without taking into account that often food can be simultaneously contaminated by more than one substance and that the contemporary exposure to mycotoxin mixtures can change the final effect exercised by the individual compounds (Benford 2017). The frequent co-occurrence of many *Alternaria* mycotoxins and the onset of additive, synergistic, and antagonistic effects have been reported in a recent study, which highlighted the need to reconsider the current risk assessment method (Crudo et al. 2019). In addition, several studies have shown that the effects exerted by a mixture of mycotoxins may vary with the concentrations of the individual mycotoxins in the mixture (Vejdovszky et al. 2017; Aichinger et al. 2019). Consequently, any factor able to modify the chemical composition of an occurring mixture could potentially modify the final toxic effect.

In this scenario, different authors reported the ability of bacteria from the human gut microbiota to metabolize mycotoxins: the *Fusarium* mycotoxin deoxynivalenol (DON) and its masked form (deoxynivalenol-3-glucoside) were found to be metabolized to de-epoxy DON and DON, respectively (He et al. 1992; Gratz et al. 2018). In addition, bacterial-mediated hydrolysis was also reported for the masked mycotoxins nivalenol-3-glucoside, T-2 toxin-glucoside, zearalenone-14-glucoside, and *α*- and *β*-zearalenol-14-glucoside (Gratz et al. 2017). The gut microbiota, as well as some food constituents, seem to be involved in xenobiotic removal processes (including mycotoxins) also through binding to the specific compounds, thus determining the reduction of their bioavailability (Boroujerdi 2015; Liew and Mohd-Redzwan 2018). Also, a recent study of our group demonstrated the ability of potentially co-ingested food constituents to spontaneously react with epoxide-carrying *Alternaria* toxins (Aichinger et al. 2018).

Considering that the gastrointestinal tract is directly exposed to mycotoxins in food, studies investigating the effects induced by *Alternaria* mycotoxins after incubation with fecal samples could provide useful information about their potential impact on health.

Therefore, the aims of the present study were to: (1) evaluate potential modifications of the genotoxic effects of an *Alternaria* mycotoxin extract on the human colorectal adenocarcinoma HT-29 cell line after 3 h of anaerobic incubation with fecal samples; (2) correlate the modifications of the genotoxic effects to changes in mycotoxin composition and concentration; (3) identify the fractions of the fecal samples mainly involved in these modifications.

## Materials and methods

### Materials

For LC–MS/MS analysis and sample preparation, methanol and acetonitrile (all LC–MS grade) were purchased from Honeywell (Seelze, Germany), while 25% ammonia solution in water and ammonium acetate (both LC–MS grade) were obtained from Sigma–Aldrich Handels GmbH (Vienna, Austria). LC–MS grade water was purchased from VWR International GmbH (Vienna, Austria). Reference materials of *Alternaria* toxins were obtained from several suppliers or were kindly provided by other researchers. For details, the interested reader may refer to Puntscher et al. (2019a).

For cell culture experiments, Dulbecco’s modified Eagle medium (DMEM), heat inactivated fetal calf serum and penicillin/streptomycin solution were purchased from Invitrogen™ Life Technologies (Karlsruhe, Germany). The human colorectal adenocarcinoma HT-29 cell line and the formamidopyrimidin‐DNA‐glycosylase enzyme (FPG) were obtained from the German Collection of Microorganisms and Cell Cultures (DSMZ, Braunschweig, Germany) and from New England Biolabs (Frankfurt, Germany), respectively.

The complex extract of *Alternaria* mycotoxins (CE) used in the present study was obtained by growing the *Alternaria alternata* DSM 62010 strain on long grain rice for 21 days, as previously described (Aichinger et al. 2019; Puntscher et al. 2019a, b). Concentrations of the *Alternaria* mycotoxins in the extract are shown in Table 1.

**Table 1:** Characterization of the used *Alternaria* extract by liquid chromatography tandem mass spectrometry (LC–MS/MS) analysis (adapted from Aichinger et al. 2019)

*Alternaria* mycotoxins	Concentration (mg toxin/g extract)
Alternariol	0.79
Alternariol monomethyl ether	0.65
Altenuene	0.78
isoAltenuene	< LOD
Tenuazonic acid	597
Tentoxin	0.02
AOH-3-glucoside	< LOD
AOH-9-glucoside	< LOD
AOH-3-sulfate	< LOD
AME-3-glucoside	< LOD
AME-3-sulfate	< LOD
Altertoxin I	9.92
Altertoxin II	14.1
Alterperylenol	12.6
Stemphyltoxin III	21.0
Altenuic acid III	< LOD
Altenusin	0.28

< *LOD* below the limit of detection

### In vitro fecal incubation

Fresh fecal samples were provided by four healthy omnivorous volunteers (i.e. two males and two females) aged 26–34 years and with a normal body mass index (18.5–24.9 kg/m^2^). No donor had previous intestinal diseases, none was treated with antibiotics, probiotics and prebiotics for the previous three months, and all signed the informed consent for the fecal sample donation. Each fecal sample was sampled with a spoon from feces on clean sampling paper, collected in a sterile plastic tube and placed in an anaerobic jar together with one anaerobiosis generator sachet (AnaeroGen™ 3.5L, Oxoid Ltd.) to keep the anaerobic conditions until their delivery to the laboratory. Samples were then transferred into an anaerobic tent (10% CO_2_, 5% H_2_ and 85% N_2_) and used within 3 h of collection.

The fecal samples were individually diluted to a concentration of 2% (w/v) with sterile phosphate-buffered saline solution (PBS, 0.1 mol/L, pH 7.4) which was previously placed into the anaerobic tent overnight to allow deoxygenation. The 2% fecal slurries (FS) obtained were then individually used for the preparation of a FS spiked with 1% dimethyl sulfoxide (FS + DMSO, used as a control) and a FS spiked with 50 µg/mL of the CE (FS + CE). The concentration of 50 µg/mL of CE was chosen to reach a final concentration of 5 µg/mL after the dilution in DMEM for producing incubation solutions, a dose which was previously reported to induce DNA damage in human cells (Aichinger et al. 2019). Five additional samples were prepared starting from the FS. Briefly, to test the effects of the soluble (sterile) part of the fecal slurry on mycotoxin stability, a filtered fecal water spiked with 1% DMSO (FW + DMSO, used as a control) and a filtered fecal water spiked with 50 µg/mL of the CE (FW + CE) were prepared by high-speed centrifugation of the FS (5200 rcf; 10 min) followed by filtration of the supernatant with 0.2 µm cellulose acetate filters and spiking. To test the effects of the particulate matter of the fecal sample, another sample (PM + CE) was obtained by low-speed centrifugation (175 rcf; 10 min) of an aliquot of the FS to pellet the particulate matter and keep most of the microorganisms in suspension (Yen et al. 2015); the pellet was then re-suspended in PBS at the same original volume and spiked with 50 µg/mL of the CE.

To investigate the contribution of living and dead microorganisms on the fate of the mycotoxins, two different aliquots of the FS were additionally centrifuged at low speed (175 rcf; 10 min) to remove the particulate matter and the supernatants were re-centrifuged at high speed (5200 rcf; 10 min) to isolate the pellet of microorganisms. One pellet was re-suspended in PBS (original volume) and spiked with 50 µg/mL of the CE (sample containing living microorganisms, LM + CE). The other pellet was re-suspended in 70% ethanol, incubated at room temperature for 5 min and centrifuged at high speed (5200 rcf; 10 min). The pellet was then re-suspended in PBS (original volume) and spiked with 50 µg/mL of the CE (sample containing dead microorganisms, DM + CE). The method of killing microorganisms by re-suspension in ethanol was adapted from Taddese et al. (2018), who demonstrated the maintenance of the cell wall integrity.

One sample of PBS spiked with 50 µg/mL of the CE was prepared for each fecal incubation and used to compare results obtained from samples containing fecal material (PBS + CE). The final concentration of DMSO was 1% (*v/v*) for all the samples prepared. Samples were then placed in glass tubes, sealed with rubber stopper to maintain anaerobic conditions, and incubated for 3 h at 37 ℃ and 150 strokes/min. Aliquots of the samples before and after 3 h of anaerobic incubation were collected and immediately stored at − 80 ℃ until the time of analysis.

### Comet assay and cell viability test

Single-cell gel electrophoresis (“comet assay”) was carried out according to the guidelines of Tice et al. (2000) with slight modifications. Briefly, 1.5 × 10^5^ HT-29 cells were seeded in Petri dishes with a diameter of 3.5 cm and grown for 48 h at 37 ℃, under humidified conditions and with 5% CO_2_. Before cell treatment, the incubated fecal samples were centrifuged at 17,000 rcf for 10 min to remove the pellet, the supernatants were diluted 1:10 with DMEM and the resulting solutions were used for incubations. PBS containing 1% (*v/v)* DMSO was diluted 1:10 with DMEM and used as a solvent control. Thus, after the dilution, the final CE concentration tested on cells was 5 µg/mL and all samples contained 0.1% DMSO. Cells were then incubated for 1 h, at the end of which a positive control was prepared by exposing cells (incubated with 0.1% DMSO) to UV-B radiation for 1 min. Then, each Petri dish was washed twice with PBS, trypsin was added to detach and singularize the cells, and cell counting and viability was carried out by trypan blue exclusion. After that, four aliquots of 30,000 cells for each sample were re-suspended, after centrifugation (420 rcf, 10 min), in 0.8% low-melting agarose and embedded on object slides (two slides for each sample). Cell lysis was carried out overnight at 4 ℃ by immersing the slides in a lysis buffer containing triton X, DMSO and N-lauryl sarcosine. One slide for each sample was treated with formamidopyrimidine–DNA glycosylase (FPG). After 30 min of incubation at 37 ℃, slides were allowed to equilibrate in an alkaline buffer (pH > 13) for 20 min, followed by electrophoresis (20 min, 25 V, 300 mA). Both, equilibration and electrophoresis were carried out on ice. Finally, the slides were washed with a neutralization buffer and stained with ethidium bromide. Microscopic analysis of DNA damages was performed using a Zeiss Axioskop (ex = 546 ± 1 nm; em = 590 nm) and the “Comet Assay IV” software (Perceptive Instruments, Suffolk, UK) was employed to score 100 cells per object slide.

### Sample preparation for MS analysis

Samples collected at the two time points were centrifuged (17,000 rcf, 15 min, 4 ℃) and the respective supernatants were diluted 1:5 with an ice-cold extraction solvent (ACN/MeOH, 1:1, v/v). Afterwards, these samples were kept at − 20 ℃ for 1 h and centrifuged again (17,000 rcf, 15 min, 4 ℃). The supernatants were then diluted 1:1 with a dilution solvent (ACN/water, 1:1, v/v) and immediately analyzed.

### LC–MS/MS analysis

Samples were analyzed using a high-performance liquid chromatographic system (HPLC, UltiMate3000, Dionex Thermo Fisher Scientific, Vienna, Austria) coupled to a TSQ Vantage triple quadrupole mass spectrometer equipped with a heated electrospray ionization (HESI) interface (Thermo Fisher Scientific). The LC–MS/MS method used in this study has been originally developed and validated for the analysis of complex food matrices (Puntscher et al. 2018) and has also been applied for the analysis of rat urine and feces (Puntscher et al. 2019b). Briefly, ammonium acetate in water solution (5 mM, pH adjusted to 8.7 with a 25% NH_4_OH solution) and MeOH were used as eluents A and B, respectively. For the chromatographic separation, a Supelco Ascentis^®^ Express C18 column (100 × 2.1 mm, 2.7 µm) equipped with a pre-column (SecurityGuard™, C18, 2 mm, Phenomenex, Torrance, CA) was used. The flow rate and the temperatures of the autosampler and the column oven were set to 0.4 mL/min, 10 ℃ and 30 ℃, respectively. The gradient was as follows: 0–1 min: 10% B, 1–1.5 min: linear increase to 38% B, 1.5–6 min: linear increase to 40% B, 6.0–6.1 min: increase to 58% B, 6.1–7.5 min: linear increase to 61%, 7.5–.0 min: linear increase to 85%, 9.0–9.1 min: increase to 100% B, 9.1–13 min: 100% B, 13.0–13.5 min: linear decrease to 10% B, 13.5–15.5 min: 10% B.

Data were acquired in multiple-reaction monitoring (MRM) mode applying negative electrospray ionization and the specific transitions for each analyte were reported by Puntscher et al. (2018). Samples were randomly analyzed, and injections of solvent blanks were routinely performed to verify the overall performance of the instrument and to avoid carry-over phenomena. Quantification of the analytes was performed by external calibration and the calibration set was injected after every 20–22 samples. Chromeleon™ Chromatography Data System Software (v. 6.80 SR13 Build 3818) and Xcalibur™ Software (v. 3.0, Thermo Scientific) were used for instrument control and data acquisition. Data evaluation was performed with TraceFinder™ (v. 3.3) Software (Thermo Scientific).

### Statistical analysis

Independent Student *t* test and analysis of variance (one-way ANOVA) with Bonferroni post hoc tests were performed using SPSS software (v. 23.0, SPSS inc., Chicago, IL, USA) to determine significant differences in relation to the two different time points of analysis and among the various samples. Samples were considered significantly different for *p* ≤ 0.05 or *p* ≤ 0.01.

## Results

### Genotoxicity

**Fig. 1:**
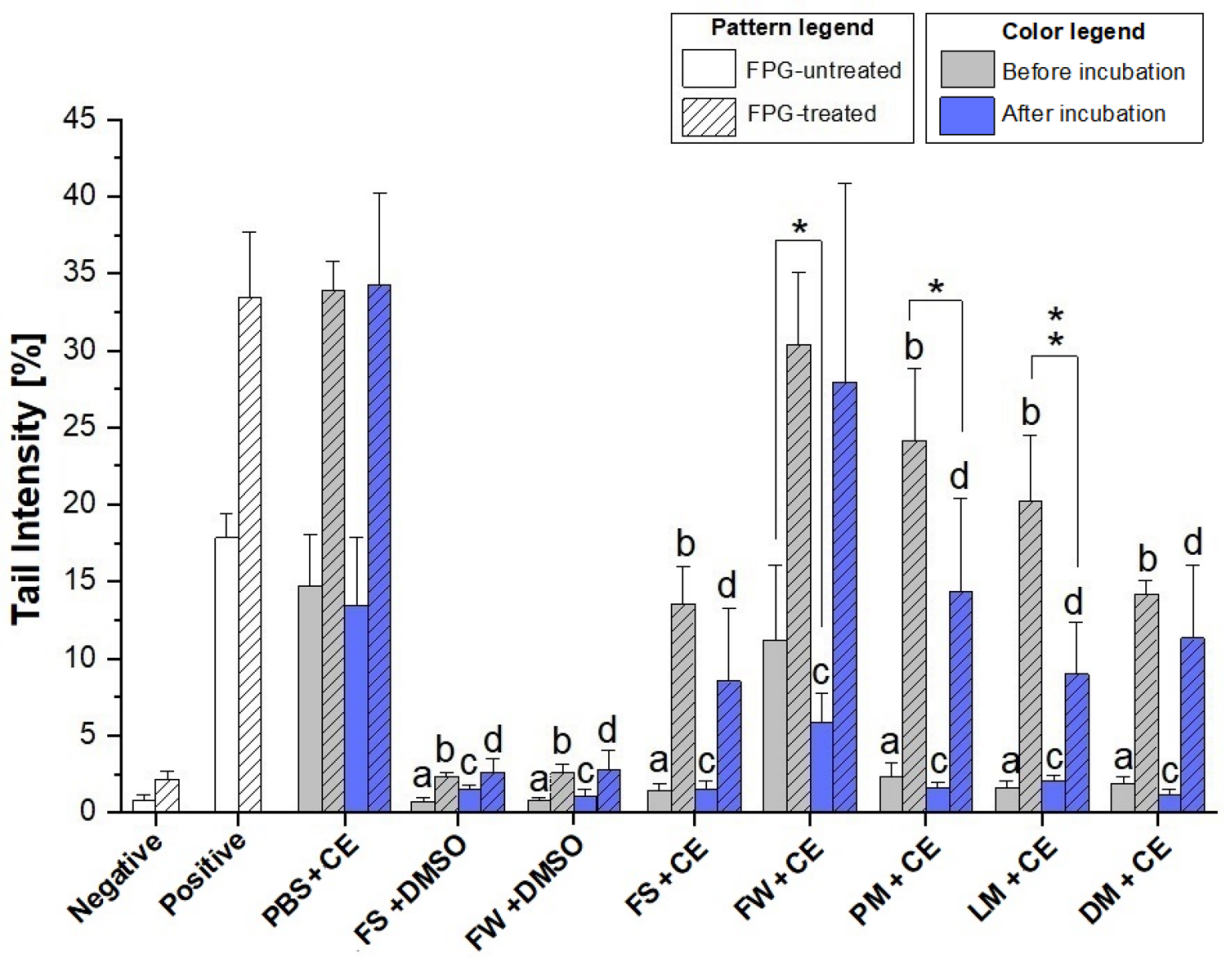
DNA strand breaks (-FPG) and additional FPG-sensitive lesions (+ FPG) measured with the comet assay in HT-29 cells after 1 h treatment with the samples collected before (0 h) and after (3 h) anaerobic incubations with fecal fractions. Cells were exposed to samples diluted 1:10 with DMEM to reach an extract concentration of 5 µg/mL. All values are expressed as mean + SD of four independent biological experiments. Significant differences between the extract in PBS (“PBS + CE”) and the other samples were calculated by one-way ANOVA, followed by Bonferroni post hoc test (*p* < 0.01), with “a”, “b”, “c”, and “d”, indicating a significant difference to the FPG-untreated PBS + CE samples before incubation, to the FPG-treated PBS + CE samples before incubation, to the FPG-untreated PBS + CE samples after incubation, and to the FPG-treated PBS + CE samples after incubation, respectively. Differences between tail intensity values found before and after anaerobic incubation for each sample were calculated by Student’s *t* test (*p < 0.05; **p < 0.01). *Negative* 0.1% DMSO, *Positive* UV light treatment, *PBS + CE* extract dissolved in PBS, *FS + DMSO* fecal slurry + DMSO, *FW + DMSO* filtered fecal water + DMSO, *FS + CE* fecal slurry + extract, *FW + CE* filtered fecal water + extract, *PM + CE* fecal particulate matter + extract, *LM + CE* living microorganisms + extract, *DM + CE* dead microorganisms + extract

Genotoxic effects of the collected samples were evaluated in HT-29 colon carcinoma cells after a dilution 1:10 with DMEM, to reach a final concentration of *Alternaria* extract on cells of 5 µg/mL. For this purpose, comet assays with or without enzymatic treatment with FPG were performed. Upon cell treatment with samples prior to anaerobic incubation, statistically significant differences (*p* < 0.01) were observed in FPG-untreated samples between the extract dissolved in PBS (PBS + CE), showing the highest tail intensity (14.7 ± 3.29%), and all the other samples containing fecal material, except for CE dissolved in fecal water (FW + CE) (Fig. 1). The mean tail intensity reached after incubation with FW + CE was slightly lower (11.2 ± 4.89%), but not significantly different compared to PBS + CE. In contrast, the other FPG-untreated samples showed tail intensities (0.72–2.30%) almost comparable to that of the negative control (0.78 ± 0.38%) (Fig. 1). FPG treatment showed the same trend, since no statistically significant differences were found between PBS + CE and FW + CE, while significantly lower tail intensities were found for all the other FPG-treated samples (*p* < 0.01) compared to PBS + CE. In this case, the recorded tail intensities of PBS + CE and FW + CE were 33.9 ± 1.88% and 30.4 ± 4.67%, respectively (Fig. 1). In contrast to what was observed for FPG-untreated samples in which the DNA-strand-break ability of mycotoxins was almost completely quenched by the presence of the fecal material, the extent of FPG-sensitive sites was mitigated but not eliminated, with tail intensities ranging from 24.1 ± 4.70% (PM + CE) to 13.5 ± 2.47% (FS + CE). Incubation with extract added to dead microorganisms (DM + CE) resulted in a lower level of FPG-sensitive sites compared to those recorded for samples containing living microorganisms, although without reaching statistical significance after Bonferroni correction.

Treatment of HT-29 cells with samples subjected to 3 h anaerobic incubation and not treated with FPG clearly showed a significant difference in mean tail intensities between the PBS + CE (13.4 ± 4.46%) and all the other samples, including FW + CE (5.82 ± 1.97%) (*p* < 0.01). Moreover, the statistical differences observed for FPG-treated samples after anaerobic incubation were similar to those observed before anaerobic incubation, although the tail intensities were different and varied from 14.3 ± 6.08% to 8.53 ± 4.75% for PM + CE and FS + CE, respectively.

Nevertheless, after fecal incubation, the tail intensities of FPG-treated samples containing dead microorganisms were found to be similar to those recorded for samples containing living microorganisms. However, a significant reduction (*p* < 0.01) of the tail intensity after 3 h of fermentation was only found in samples of living microorganisms compared to the respective samples before fermentation.

To exclude any intrinsic genotoxic activity of the fecal material, which could have distorted the results obtained from the samples containing the extract, FS + DSMO and FW + DMSO control samples from each donor were also analyzed whereby no enhanced levels of DNA strand breaks or FPG-sensitive sites were observed.

With regard to differences observed before and after anaerobic incubation, a significant decrease (*p* < 0.05) of the DNA-strand break properties was only observed for complex extract in fecal water (FW + CE). With respect to FPG-sensitive sites, anaerobic incubation in the presence of particulate matter or living microorganisms showed significant mitigative effects (*p* < 0.05 and *p* < 0.01, respectively), while samples prepared by the use of dead microorganisms (DM + CE) did not affect the level of DNA damage.

### Cell viability

To exclude artefacts deriving from cytotoxic effects, potentially induced by the mycotoxins or by the fecal material, cell viability was determined by trypan blue exclusion test prior to the comet assay. After 1 h of treatment of HT-29 cells with the collected samples, the resulting cell viabilities were all above 80% and ranged from 89.8 ± 1.87% to 97.1 ± 2.22% for the samples before anaerobic incubation and from 90.5 ± 1.36% to 94.5 ± 0.98% for the samples collected after 3 h of anaerobic incubation. Therefore, these results confirmed that the genotoxic effects observed were directly ascribable to the genotoxic properties of the mycotoxins contained in the extract and not to cytotoxicity phenomena (Online Resource 1).

### Impact on mycotoxin prevalence

Mycotoxins contained in the original *Alternaria* extract were quantified by LC–MS/MS in the samples collected after 0 h and 3 h of anaerobic incubation to identify any possible modification induced by the presence of fecal material. In particular, eight different mycotoxins were quantified: AOH, AME, TeA, TEN, ATX-I, ATX-II, AST, and ALP. The mycotoxin STTX-III was also monitored, but not quantified due to limitation of reference material.

**Fig. 2:**
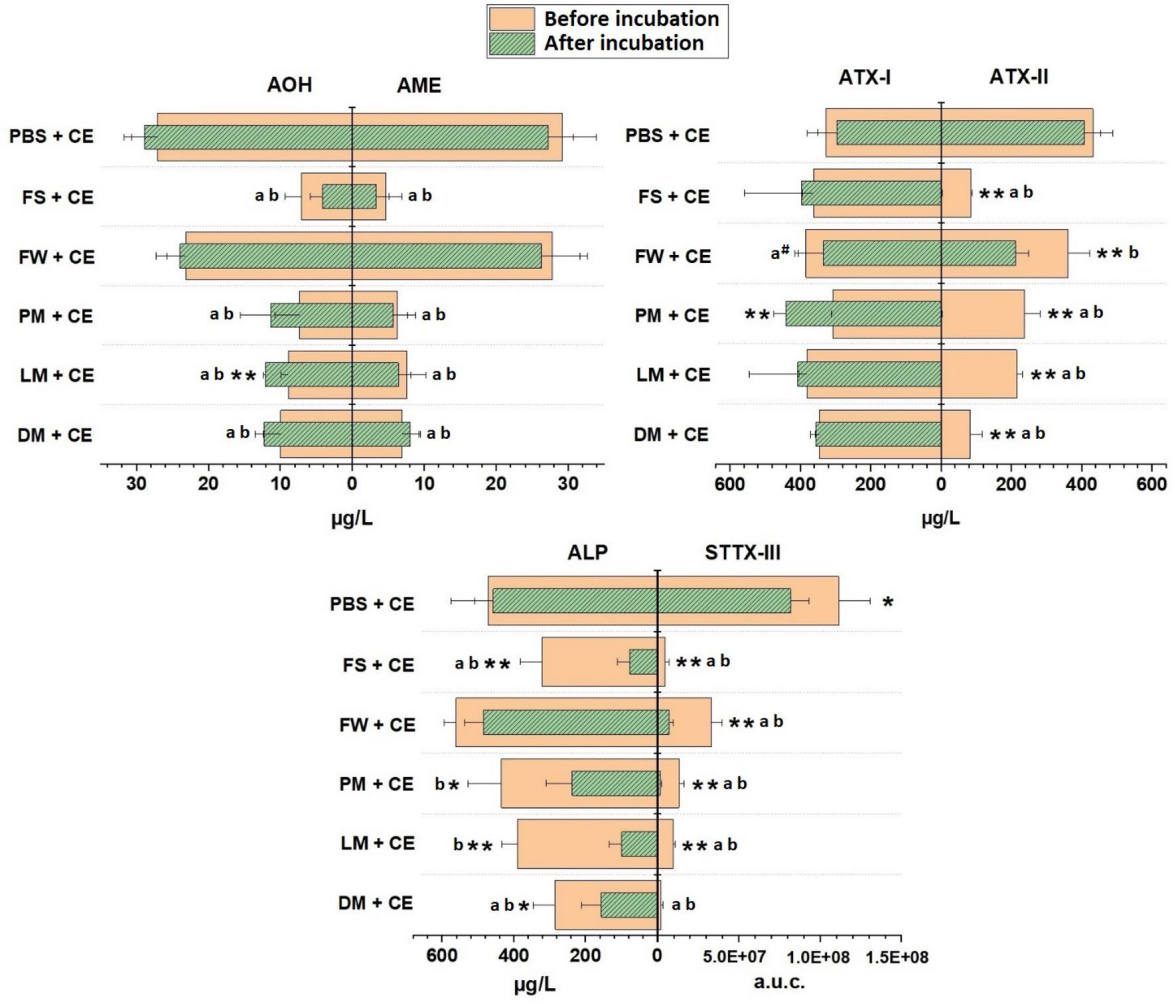
Bar charts highlighting mycotoxins affected by contact with fecal material. All values are expressed as mean + SD of four independent biological experiments. Differences between concentrations found before and after anaerobic incubation for each sample were calculated by Student’s *t* test (*p < 0.05; **p < 0.01). Significant differences between mycotoxin concentrations found in the extract-containing PBS control (“PBS + CE”) and the other samples were calculated by one-way ANOVA, followed by Bonferroni post hoc test (*p* < 0.01). **a** Significant difference between the mycotoxin concentration found in samples containing fecal material before incubation and the PBS + CE control before incubation; **b** significant difference between the mycotoxin concentration found in samples containing fecal material after incubation and the PBS + CE control after incubation. Letters followed by “#” indicate a significant difference at *p* < 0.05. *PBS + CE* extract dissolved in PBS, *FS + CE* fecal slurry + extract, *FW + CE* filtered fecal water + extract, *PM + CE* fecal particulate matter + extract, *LM + CE* living microorganisms + extract, *DM + CE* dead microorganisms + extract, *a.u.c.* area under the curve

The blank control samples (FS + DMSO and FW + DMSO) were analyzed to ensure the absence of pre-existent *Alternaria* mycotoxins and they were found to be negative (< LOD) for all the mycotoxins under investigation. With regard to the extract-containing samples, the mean concentrations of the mycotoxins that were strongly affected by the presence of fecal material are reported in Fig. 2, while the data from the unaffected mycotoxins are shown in Table 2.

**Table 2:** Mean concentrations of *Alternaria* mycotoxins unaffected by fecal incubation in samples spiked with 50 µg/mL of the extract

Sample	Mean concentration											
	TeA [mg/L]				TEN [µg/L]				AST [µg/L]			
	T 0 h		T 3 h		T 0 h		T 3 h		T 0 h		T 3 h	
PBS + CE	20.8	± 4.26	24.0	± 0.69	0.91	± 0.48	0.65	± 0.42	227	± 71.9	149	± 36.9
FS + CE	21.5	± 2.80	23.8	± 6.84	0.61	± 0.18	1.01	± 0.21*	125	± 23.0	92.6	± 49.5
FW + CE	24.4	± 1.50	18.5	± 9.73	0.68	± 0.28	0.71	± 0.21	357	± 64.1	350	± 78.3
PM + CE	17.3	± 4.97	24.6	± 3.58	0.63	± 0.28	0.89	± 0.13	195	± 43.3	153	± 53.3
LM + CE	26.7	± 1.58	21.9	± 5.02	1.34	± 0.42	0.75	± 0.43	151	± 32.7	127	± 36.2
DM + CE	24.6	± 1.64	23.1	± 3.07	1.11	± 0.31	0.95	± 0.46	189	± 32.1	180	± 26.6

All values are expressed as mean ± SD of data obtained from the four donors

*PBS* + *CE* extract dissolved in PBS, *FS* + *CE* fecal slurry + extract, *FW* + *CE* filtered fecal water + extract, *PM* + *CE* fecal particulate matter + extract, *LM* + *CE* living microorganisms + extract, *DM* + *CE* dead microorganisms + extract

*Indicates a significant difference (*p* < 0.05) compared to the respective time point 0

Among the various *Alternaria* toxins, AOH concentrations were not affected by the presence of the soluble fraction of the feces (fecal water, FW), since no difference in its content was found between FW + CE and PBS + CE samples. Nevertheless, AOH concentrations decreased considerably already without anaerobic incubation by the addition of fecal particulate matter, living or dead microorganisms (*p* < 0.01). Additionally, AOH concentrations were significantly higher in the samples containing living microorganisms after 3 h of incubation than those recorded before incubation (*p* < 0.01).

AME concentrations in samples before anaerobic incubation showed the same behavior as AOH, without any modification in FW + CE compared to PBS + CE and with a significant decrease (*p* < 0.01) in the other samples. In contrast to what was observed for AOH, the anaerobic incubation period did not affect the AME content of all the samples tested.

Among the quantified mycotoxins, ATX-II showed the highest reduction in samples containing fecal material. This was probably because of its high reactivity deriving from the reactive epoxy group present in its chemical structure which made the mycotoxin prone to react with the substances present in fecal samples. Although its presence in PBS + CE and FW + CE was the same before anaerobic incubation (Fig. 2), the concentrations were strongly diminished (*p* < 0.01) in all the other samples, with a maximal reduction of 80.6% in FS + CE compared to PBS + CE. A similar percentage of reduction was observed by the addition of dead microorganisms (DM + CE), whereas addition of living microbes reached only a reduction of 50.2%. After anaerobic incubation, ATX-II was not detected (< LOD) any more in FS + CE, PM + CE, LM + CE and DM + CE, and a reduction of its concentration (47.8%) was found in fecal water samples compared to PBS + CE. In addition, comparing the concentrations of ATX-II found in FW + CE samples at the two time points, a reduction of 41.0% of its content was found after anaerobic incubation. ALP was one of the most stable mycotoxins before anaerobic incubation, since reductions in its concentration were found only in FS + CE (− 31.6%; *p* < 0.05) and DM + CE (− 39.7%; *p* < 0.01). Different results were obtained for the samples subjected to anaerobic incubation: ALP concentrations remained stable in FW + CE, while reductions of 48.1–83.2% were observed in the other samples. Differences between the two time points were found in all the samples tested except for FW + CE. Although the quantification of STTX-III was not possible, the graph reported in Fig. 2 highlights the tendency of the mycotoxin to decrease, already before anaerobic incubation, in all the samples containing fecal material compared to the control PBS + CE. In contrast to the other measured mycotoxins, an evident reduction of the STTX-III peak size was found also in FW + CE and, at the same time, its instability was even more evident in the other samples. After 3 h incubation, nearly a total loss of STTX-III was detected in all the samples, and a significant reduction of its content (*p* < 0.05) was also found in the control PBS + CE.

Among all the mycotoxins measured, TeA, TEN and AST showed the highest stability (Table 2), since their concentrations were mostly unaffected by the addition of fecal material and by anaerobic incubation. In particular, no difference in TeA concentrations was found in samples containing fecal material compared to the control PBS + CE. However, TeA concentrations were slightly lower (*p* < 0.05) in PM + CE samples before anaerobic incubation compared to LM + CE at the same time point. In addition, TeA content in the sample set did not show any variation over the time. Similarly, TEN and AST concentrations were unaffected by the presence of the fecal material before incubation and their stability was even more evident in the samples after 3 h of incubation, since no significant difference was found comparing the two time points of each sample. However, AST concentrations in FW + CE samples before and after anaerobic incubation were higher than those found in PBS + CE samples.

Unlike for ATX-II, ATX-I concentrations were almost the same in all the tested samples before anaerobic incubation, except for FW + CE samples that were characterized by a higher amount of ATX-I compared to PBS + CE (p < 0.05). Interestingly, although after 3 h of anaerobic incubation, no difference was found in ATX-I concentrations among the samples, a higher concentration of ATX-I was determined in PM + CE samples compared to the respective concentrations before incubation (p < 0.01). In addition, the FS + CE samples of 3 out 4 donors showed comparably high levels of ATX-I after the anaerobic incubation (457–507 µg/L), in contrast to a low concentration found in one donor only (155 µg/L). This low concentration of ATX-I found in the sample of the fourth donor was responsible for the failure to achieve a statistically significant difference between the mean ATX-I concentrations found before and after the anaerobic incubation of the FS + CE samples.

## Discussion

*Alternaria* mycotoxins are frequently found to co-occur in different food matrices and their potential to exert harmful effects has been already reported in several in vitro and in vivo studies. However, limited information is available about the possible influence of both the gut microbiota and co-digested food constituents on the bioavailability and bioactivity of the mycotoxins, and therefore about the possible modifications of the overall toxicity. To evaluate whether the different fractions of fecal samples modify the genotoxicity of a complex mixture of *Alternaria* mycotoxins, comet assays were performed. A preliminary investigation to exclude possible intrinsic genotoxic properties of the fecal material was carried out and data confirmed the direct genotoxic activity of the extract, whereas in the absence of the extract, no DNA strand breaks or enhanced levels of FPG-sensitive sites were induced by samples of fecal slurry or fecal water in the applied concentrations. With regard to the samples containing the *Alternaria* extract, results from the comet assay clearly showed a limited impact of the presence of fecal material or even anaerobic incubation on the induction of FPG sensitive sites. These results might argue for the persistence of pro-oxidative properties of the *Alternaria* extract, but might also reflect other FPG-sensitive lesions. Among the mycotoxins contained in the extract, the *Alternaria* mycotoxins AOH and AME were found to induce oxidative DNA damage and reduce intracellular levels of the antioxidant glutathione in HT-29 cells already after 1 h of incubation (Tiessen et al. 2013). However, authors reported the disappearance of oxidative DNA damage after 3 h of incubation, probably due to inactivation of the compounds or enhanced DNA repair. In contrast to what was observed for the FPG-sensitive sites, the DNA strand breaks induced by the extract were completely quenched by the presence of microorganisms, particulate fecal matter or the whole fecal slurry. Genotoxic properties were only maintained in the presence of fecal water supernatants (Fig. 1). These results are supported by LC–MS/MS analysis, since the concentrations of most of the mycotoxins were reduced in samples containing the particulate matter of the fecal samples, living or dead microorganisms (Fig. 2). On the contrary, fecal water spiked with the extract did not show any significant difference in the mycotoxin pattern compared to the extract in PBS, thus excluding a possible role of the soluble proportion of the fecal samples in the observed modifications. Only STTX-III showed a very high instability even in the samples containing fecal water. In addition, this instability was also clearly evidenced by the significant reduction of STTX-III observed in the extract-containing PBS samples (“PBS + CE”) after 3 h of anaerobic incubation (Fig. 2). These results may be explained by the presence of the reactive epoxy group in its chemical structure. STTX-III, in fact, was previously reported to be instable even in solvent solutions (Zwickel et al. 2016). However, the involvement of STTX-III in esophageal cancer pathogenesis cannot be completely excluded, as suggested before (Zwickel et al. 2016). Furthermore, there is a lack of knowledge on potential degradation products of STTX-III or closely relate perylene quinones, such as ATX-II, which might exert toxicological effects themselves.

Among the tested *Alternaria* mycotoxins, TeA, TEN, AST and ATX-I showed the highest stability in samples containing the various fecal fractions. Thus, considering the nearly unmodified concentrations of these mycotoxins in the samples containing fecal fractions compared to the extract in PBS and the decrease of DNA-strand-breaking properties observed already without further anaerobic incubation, TeA, TEN, AST and ATX-I seem to be not involved in DNA damage induced by the complex extract. Our results are in accordance with literature describing TeA and TEN as not genotoxic (EFSA 2011; Schwarz et al. 2012a). On the contrary, ATX-I, albeit being less potent than ATX-II, was reported to induce DNA strand breaks in human and animal cancer cell lines (Fleck et al. 2014), while, no information is currently available about possible genotoxic properties of AST. However, in the present work, no DNA strand breaks were associated with ATX-I presence and this may be a consequence of the very low final concentration the HT-29 cells that were exposed to in the comet assay (0.08 µM). Nevertheless, it is important to highlight that the presence of substances exerting other kinds of genotoxic effects, e.g. oxidative DNA damage, cannot be completely excluded (e.g. bioactive compounds deriving from the rice used for the growth of the *Alternaria alternata* strain or yet undiscovered mycotoxins).

Considering our results, the soluble part of the fecal slurry played a marginal role in attenuating the genotoxicity of the extract. This observation was directly related to the unmodified concentrations of most of the mycotoxins prior and after anaerobic incubation (Table 2 and Fig. 2). This applies to both DNA strand breaks and FPG-sensitive sites before anaerobic incubation, while it applies only to the FPG-sensitive sites for the samples after 3 h of incubation. As a matter of fact, a significant reduction of DNA strand breaks was observed in samples of fecal water after anaerobic incubation and it was mainly related to the significant losses of ATX-II and STTX-III, whose abilities to induce DNA strand breaks have been recently demonstrated (Fleck et al. 2016; Jarolim et al. 2017). The loss of ATX-II was probably due to reactions of the mycotoxin with constituents present in the fecal water samples, while the loss of STTX-III might be attributed both to reaction phenomena with soluble unabsorbed substances and to a lack of stability of the mycotoxin over time, since a decrease of its concentration was observed even in the extract dissolved in PBS (“PBS + CE”) after 3 h of anaerobic incubation.

The decrease of ATX-II content could be, in turn, a consequence of its reduction to ATX-I. The epoxy bearing moiety of ATX-II was previously reported to be prone to reactions with thiol moieties, which might result in the formation of ATX-I (with dithiols) or monothiol adducts of ATX-II (with monothiols) (Fleck et al. 2014). The proposed mechanism for the reduction of ATX II to ATX-I by dithiols was based on the initial formation of a monothiol adduct (with one of the two thiol groups of the dithiol compound), followed by the formation of five- or six-membered rings (depending on the type of dithiol compound). Thus, the second thiol group would be responsible for an intramolecular reduction reaction that leads to the formation of ATX-I and the oxidized form of the dithiol. Transformation of ATX-II in ATX-I was also reported in tomatoes during storage, as well as in biological samples of rats after oral application of ATX-II (Puntscher et al. 2019a, c). Despite not yet elucidated, this mechanism could at least contribute to the loss of STTX-III observed in the present study.

The slightly higher levels of ATX-I found in samples of fecal water (FW + CE) before anaerobic incubation compared to the respective control in PBS could be the consequence of the previously described mechanism involving the reaction of ATX-II with the soluble substances of the feces. Additionally, the absence of microorganisms and insoluble fecal matter in these samples, which might have partially adsorbed the toxin, prevented from underestimating the real content. Higher concentrations of ATX-I, together with a total loss of ATX-II, were also observed after anaerobic incubation in samples containing the particulate matter of the fecal samples (“PM + CE”). Despite the presence, in this case, of the particulate matter, the increase in ATX-I was not completely masked by adsorption phenomena.

Adsorptive effects exerted by different compounds and structures and involving xenobiotics have been widely reported in literature. The *Alternaria* mycotoxins AOH, AME and ALT (Lemke et al. 2016), ochratoxin A (Kabak et al. 2009) and the aflatoxins B_1_, B_2_, G_1_ and G_2_ (Kabak and Ozbey 2012) were reported to be adsorbed to the bacterial cell surface. In addition, other studies highlighted the ability of many agricultural products and by-products, especially when rich in nondegradable dietary fibers, to adsorb xenobiotics introduced with the diet, thus preventing the onset of toxicosis. As an example, grape pomace was found to sequester different mycotoxins, including aflatoxin B_1_, zearalenone, ochratoxin A, fumonisin B_1_, and, moderately, also deoxynivalenol (Avantaggiato et al. 2014). Similarly, different food plants were able to adsorb mycotoxins potentially occurring in the normal diet and the ability of indigestible fibers to contrast the toxic effects induced by some mycotoxins was also reported in various in vivo studies (Greco et al. 2019). All these findings support the results obtained in the present work; already before anaerobic incubation, the concentrations of most of the mycotoxins tested (AOH, AME, ATX-II, STTX-III and ALP) were lower in the samples containing microorganisms, particulate fecal matter (which is supposed to be composed also by indigestible fiber fractions), and both of them (“FS + CE”), compared to the matrix-free toxin mixture in PBS (Fig. 2).

The maximum level of mitigation of the genotoxic properties of the studied complex *Alternaria* extract, as well as the maximum reduction of the original mycotoxin concentrations, was recorded in samples prepared with the whole fecal slurry (“FS + CE”). This finding suggests a cooperation of the different fractions of the feces (particulate matter and microorganisms) in the “detoxification” processes. However, it has to be pointed out that the reduction of genotoxicity of this natural mixture of *Alternaria* toxins predominantly related to an effective suppression of the direct DNA-strand-breaking potential, while the potency to induce FPG-sensitive sites remained largely unaffected, raising the question on the relevant extract constituents. Since a natural extract of an *Alternaria* culture was applied, it has to be underlined that not all constituents have been elucidated yet and it cannot be excluded that so far unknown constituents play a role for the persistent induction of FPG-sensitive sites. From the already identified constituents, ALP is a potential candidate with its concentrations largely unmodified before anaerobic incubation in almost every samples, and showing a reduction only after 3 h of incubation (except in samples of fecal water). Anyway, the presence of a considerable amount of ALP even after the incubation may pose the question whether this mycotoxin might be involved in the overall genotoxicity of the *Alternaria* extract, since no information concerning its genotoxic properties is available to date.

With regard to the effects exerted by living microorganisms, in the present study, no direct correlation between the metabolic activity of the gut microbiota and the *A.* mycotoxin patterns with their genotoxic potentials was observed. The only data suggesting a possible role of microorganisms in the modifications of mycotoxin concentrations are related to the mycotoxin AOH. In fact, AOH concentrations were significantly higher in samples containing living microorganisms after 3 h of incubation than those recorded before incubation (*p* < 0.01). However, it has to be pointed out that the significant difference found was due to the very low standard deviation value of samples containing living microorganisms after 3 h of anaerobic incubation. In our opinion, this finding cannot be related to the metabolic activity of microorganisms, also considering that no difference was found in AOH concentrations among samples containing particulate matter, living and dead microorganisms.

To date, only one study focused on the evaluation of the ability of gut microbiota microorganisms to modify the *Alternaria* mycotoxins AOH, AME and ALT (Lemke et al. 2016), and no information is currently available about the other *Alternaria* toxins contained in the extract used in this work. Considering that results related to not-yet-investigated *Alternaria* mycotoxins have been also reported for the first time in the present work, and that these results refer to the effects induced by short-term fecal incubations, further studies dealing with the evaluation of a possible involvement of gut microbiota in mycotoxins metabolization in long-term fecal incubations should be encouraged for further clarification. However, it is difficult to design such experiments since they would require the use of growth media to keep the microorganisms of the gut microbiota alive and metabolically active. Growth media might in fact alter the initial microbial composition of the feces, favoring the growth of some microorganisms at the expense of others. Anyway, the results obtained in the present work represent a starting point, suggesting that adsorptive phenomena of genotoxic *Alternaria* mycotoxins to bacterial cells may have occurred.

## Conclusion

This is the first in vitro study investigating the effects of an anaerobic fecal incubation on the genotoxic effects induced by a complex mixture of *Alternaria* mycotoxins. We observed the ability of both, microorganisms and undigested food constituents to suppress the DNA-strand-breaking potential induced by the applied extract, while the potency to induce FPG-sensitive lesions remained largely unaffected. These results were related to an observed reduction of mycotoxin concentrations, especially those of alternariol and its monomethyl ether, altertoxin II, stemphyltoxin III and alterperylenol.

Although a direct correlation between the metabolic activity of the gut microbiota and the modifications in mycotoxin content was not found, possible adsorptive phenomena of mycotoxins to the bacteria cells and food constituents may explain these results.

In the light of this, the present study provides useful data for assessing the risk related to the multitude of tested *Alternaria* mycotoxins, suggesting that their toxic effects might be quenched or reduced by the digestive process and by the intestinal environment. Subsequent studies are needed to explore the fate of toxins by untargeted high-resolution MS approaches to investigate the type of interactions determining the loss of mycotoxins, as well as the actual role of the gut microbiota in the metabolization of the *Alternaria* mycotoxins.

## Electronic supplementary material

Below is the link to the electronic supplementary material.

Supplementary file1 (DOCX 36 kb)

## Abbreviations

AOH: Alternariol
AME: Alternariol monomethyl ether
ALT: Altenuene
TeA: Tenuazonic acid
TEN: Tentoxin
ALP: Alterperylenol
AST: Altersetin
ATX: Altertoxin
STTX-III: Stemphyltoxin III
FPG: Formamidopyrimidin‐DNA‐glycosylase enzyme
CE: Complex extract of *Alternaria* mycotoxins
