# Supplementary material for: Gut microbiota and undigested food constituents modify toxin composition and suppress the genotoxicity of a naturally occurring mixture of Alternaria toxins in vitro
Source: Arch Toxicol. 2020 Jul 4;94(10):3541–52. doi: 10.1007/s00204-020-02831-1 (PMC7502057; doi:10.1007/s00204-020-02831-1)
Supplement: Supplementary file 1 — Supplementary file1 (DOCX 36 kb) [file 204_2020_2831_MOESM1_ESM.docx]

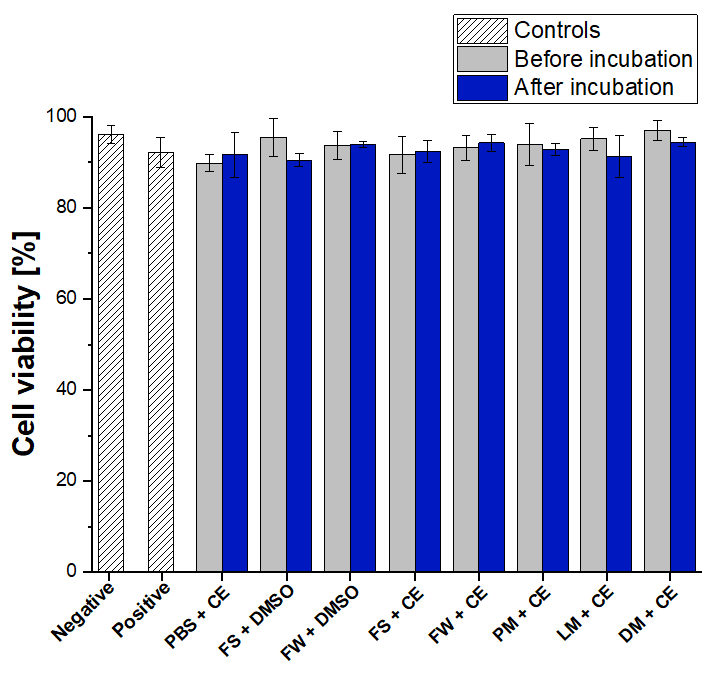


**Online Resource 1** Impact of the samples collected before (0 h) and after (3 h) anaerobic incubation with fecal fractions on the viability of HT-29 cells, as measured through trypan blue exclusion test after 1 h incubation. Cells were exposed to samples diluted 1:10 with DMEM to reach an extract concentration of 5 µg/ml. All values are expressed as mean ± SD of data obtained from the four donors.

Abbreviations: “Negative”= 0.1% DMSO; “Positive”= UV light treatment; “PBS+CE”= extract dissolved in PBS; “FS+DMSO”= fecal slurry + DMSO; “FW+DMSO”= filtered fecal water + DMSO; “FS+CE”= fecal slurry + extract; “FW+CE”= filtered fecal water + extract; “PM+CE”= fecal particulate matter + extract; “LM+CE”= living microorganisms + extract; “DM+CE”= dead microorganisms + extract.
